# Molecular and Virological Investigation of a Focal Chikungunya Outbreak in Northern India

**DOI:** 10.1155/2013/367382

**Published:** 2013-12-24

**Authors:** Manisha Soni, Anil Kumar Singh, Shashi Sharma, Ankita Agarwal, Natarajan Gopalan, P. V. Lakshmana Rao, Manmohan Parida, Paban Kumar Dash

**Affiliations:** Division of Virology, Defence R&D Establishment, Jhansi Road, Gwalior 474002, India

## Abstract

Chikungunya (CHIK) fever is one of the most important arboviral infections of medical significance. The objective of the present study is to identify and characterize the etiology of a focal febrile arthritis outbreak from Gwalior, northern India, during October-November 2010. A detailed virological (isolation) and molecular (end-point RT-PCR, quantitative RT-PCR, and nucleotide sequencing) investigation of this outbreak was carried out by collecting and studying 52 clinical samples and 15 mosquito pools from the affected region. The investigation revealed the presence of CHIK viral RNA in 29% of clinical samples and 13% mosquito pool by RT-PCR. The quantification of CHIK viral RNA in samples varied from 10^2.50^ to 10^6.67^ copies/mL, as demonstrated through quantitative RT-PCR. In addition, six CHIK viruses were isolated from RT-PCR positive samples. The nucleotide sequences of partial E1 gene of five representative CHIK viruses were deciphered, which revealed that all the viral strains from this outbreak belong to the recently emerging ECS African genotype. Identification of Chikungunya virus ECSA African genotype as the etiology of the present outbreak confirms the continued circulation of the novel genotype, since 2006, in India. The identification of CHIK virus in *Aedes aegypti* also confirmed it as the major vector in northern India.

## 1. Introduction

Chikungunya fever has emerged as one of the most important arboviral infection of public health significance. It is endemic in many parts of Africa and Asia. Since January 2005, many countries in the Indian Ocean and Asia witnessed unparallel Chikungunya outbreaks [1, 2]. Chikungunya virus (CHIKV), the etiological agent, belongs to the family *Togaviridae* and the genus *Alphavirus*. CHIKV is primarily transmitted by *Aedes aegypti *and* Aedes albopictus *mosquitoes and is maintained through a man-mosquito-man cycle in nature [3]. Chikungunya infection in human is characterized by abrupt clinical onset, involving fever, headache, fatigue, nausea, myalgia, and severe arthralgia. This is followed by constitutional symptoms that include maculopapular rash on the trunk and limbs. Symptoms are generally self-limiting and can last for around a week, although arthralgia may persist in a small proportion of cases even for months [4, 5]. However, during recent outbreaks, cases involving unusual manifestations and severities, including neurological complications and mortality, have been widely reported [1, 6]. Currently, there is no specific vaccine or antiviral therapeutics available against Chikungunya infection.

The genome of CHIKV consists of a linear, single stranded, positive sense ribonucleic acid (RNA) of approximately 11.7 kb in length [7]. CHIKV was first isolated from Tanzania, Africa, in 1953. Later, it has caused numerous outbreaks in continental Africa, the Indian Ocean region, and Southeast Asia including India. It was first reported in 1963 from Calcutta. The reemergence of Chikungunya outbreaks is generally unpredictable and occurs frequently after 8–20 years of silence [8]. The resurgence of Chikungunya after 2005 is considered unprecedented, owing to the geographical expansion and magnitude of morbidity. Chikungunya has resurged in the form of explosive epidemic in India, in 2006, after a gap of 32 years [9]. Since then, the disease continued in many parts of India affecting more than 4 million persons till 2010, causing huge public health concern [10]. Molecular investigation of these outbreaks revealed emergence of a new variant African genotype of CHIKV as the etiology [11–13]. In the present study, we have carried out a detailed molecular investigation by collecting both human and mosquito samples from affected area of Gwalior, Madhya Pradesh, and northern India to identify and characterize the etiology of this focal outbreak during October-November 2010.

## 2. Materials and Methods

### 2.1. Clinical Samples

**Figure 1:**
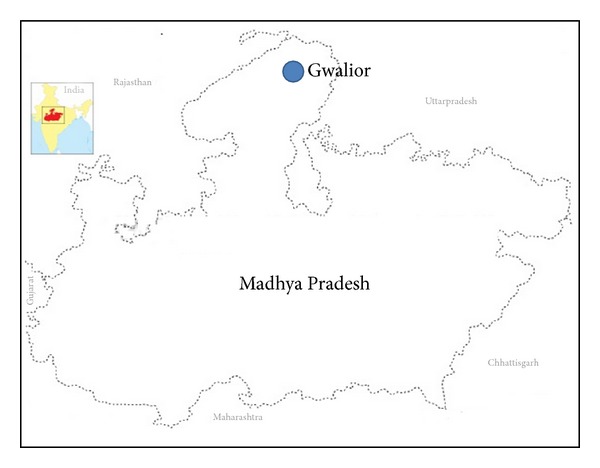
Map of the state of Madhya Pradesh, India, showing the location of Chikungunya affected area.

A total of 52 blood samples from clinically suspected Chikungunya patients were collected from G. R. Medical College, Gwalior, Madhya Pradesh and Government PHC, Atri, Gwalior, MP, during October-November 2010 (Figure 1). Informed consent was obtained from all the patients prior to sample collection. Serum was separated from the blood samples and stored at −80°C till use.

### 2.2. Mosquito Samples

Fifteen adult *Aedes aegypti *mosquito pools (size ranging from 8 to 30) were collected from eight different locations in the village of Atri, and the city of Gwalior. The adult pools were frozen at −80°C till use.

### 2.3. Reverse Transcription-Polymerase Chain Reaction (RT-PCR)

The RNA from serum samples and clarified homogenate of mosquito pools were isolated using QIAamp viral RNA minikit (Qiagen, Germany) in accordance with the manufacturer's instructions. The presence of CHIKV specific RNA was detected using Enhanced Avian HS RT-PCR kit (Sigma, USA) employing a primers pair targeting the E1 gene (CHIK13: TTACATCACGTGCGAATAC genome position 10128–10146 and CHIK14: CTTTGCTCTCAGGCGTGCGACTTT genome position 10604–10627), designed from the nucleotide sequence of the reference S27 strain, GenBank Acc no. AF490259. The thermal profile for RT-PCR was a reverse transcription step at 48°C for 45 min and denaturation at 95°C for 3 min, followed by 35 cycles of thermal cycling, which included denaturation at 95°C for 1 min, annealing at 55°C for 1 min, an extension at 72°C for 1 min and final extension at 72°C for 10 minute. The amplicons were verified by 1.5% standard agarose gel electrophoresis.

### 2.4. SYBR Green-Based Quantitative RT-PCR (qRT-PCR)

The quantification of Chikungunya viral RNA in clinical samples and mosquitoes was carried out employing a one-step real-time SYBR Green-based qRT-PCR [14]. Briefly, qRT-PCR was carried out in a final volume of 25 *μ*L using SuperScript III Platinum SYBR Green One-Step qRT-PCR kit (Invitrogen, USA) containing 12.5 *μ*L of 2X SYBR green reaction mix, 1 *μ*L of RT/Taq mix, and 10 *μ*mol each of CHIKV specific sense and antisense primers (CK1: ACGCAATTGAGCGAAGCAC and CK2: CTGAAGACATTGGCCCCAC) in Mx3005 instrument (Stratagene, USA). The thermal profile was RT step at 50°C for 10 min, followed by holding at 95°C for 5 min, and then 40 cycles of denaturation at 95°C for 15 s, annealing at 55°C for 30 s, and extension at 72°C for 30 s. Following amplification, a melting curve analysis was performed to verify the authenticity of the amplification. A standard curve was drawn using 10-fold serially diluted *in vitro* transcribed RNA, as described earlier [15]. The RNA copies were determined from the respective Ct value obtained from the standard curve.

### 2.5. Virus Isolation

The PCR positive acute phase serum samples and clarified homogenate of mosquito samples were passaged in C6/36 for isolation of the virus. Briefly, culture tubes (Nunc, Denmark) containing preformed monolayer of C6/36 cells were adsorbed with 0.2 mL of plasma samples (diluted 1 : 10 in EMEM and filtered through membrane of 0.22 *μ* pore diameter) for 90 min at 37°C with intermittent shaking. The inoculum was then replenished with 2 mL of maintenance medium (EMEM with 2% FBS). Suitable cell controls were also kept alongside. The cells were harvested on appearance of cytopathic effects or on the fourth day after infection (dpi), which ever occurred earlier. Each sample was passaged thrice before declaring as negative. The Identification of the virus isolates obtained from the clinical samples was carried out by RT-PCR.

### 2.6. Nucleotide Sequencing and Phylogenetic Analysis

Double pass sequencing of five CHIK viruses (four from clinical samples and one from mosquito pools) was carried out with a Big dye terminator cycle sequencing ready reaction kit (Applied Biosystems, USA) on an ABI3130 sequencer following the manufacturer's protocol. Following sequencing, the nucleotide sequences were edited and analyzed with the *EditSeq* and *MegAlign* modules of the Lasergene 5 software package (DNAStar Inc., USA). Phylogenetic analysis was conducted using MEGA version 5.03 [16]. Tamura Nei model of nucleotide substitution with gamma-distribution rates available in MEGA was used to construct the Neighbor-Joining tree. The tree topologies were evaluated using 10000 replicates of the data set.

## 3. Results

### 3.1. The Epidemic

A focal outbreak of febrile arthritis was reported in Gwalior region of Madhya Pradesh and some other parts in the central India in October-November 2010, affecting a large population in the area. A total of 52 clinical samples were collected from the affected area. The clinical history revealed that all patients had suffered from acute onset of fever with headache and joint pain. Common symptoms were fever with chill, joint pain, and headache. Anorexia, nausea, and abdominal pain were the other associated minor symptoms (Table 1). The majority of the patients have severe joint pain with swelling. The sex ratio of the infected individuals was 3 : 2 (male : female). The maximum individuals infected were found to be in the age group of 24–40 years.

### 3.2. RT-PCR

A total of 15 (29%) clinical samples were found to be positive for the presence of CHIKV specific RNA, through demonstration of CHIKV specific 500 bp amplicons on agarose gel. Similarly, two (13%) adult *Aedes aegypti* mosquito pools were found positive for CHIKV RNA.

### 3.3. SYBR Green-Based Quantitative RT-PCR (qRT-PCR)

The quantification of CHIKV RNA in clinical samples varied from 10^2.50^ to 10^6.76^ copies/mL. The average CHIKV RNA titre in adult *Aedes aegypti* mosquito pools was 10^3.09^ copies/mL. The result revealed one additional positive sample (DRDE10_GWL9), compared to conventional RT-PCR (Table 2).

### 3.4. Virus Isolation

The three serial passages of 15 RT-PCR positive clinical samples resulted in isolation of six CHIKV isolates. Virus isolation was not successful from mosquito samples. The cytopathic effect was characterized by an irregular morphology and foamy degeneration of cells at 3-4 dpi. The mock infected cells remained healthy, with no changes in their cellular morphology. The isolations were also confirmed by RT-PCR.

### 3.5. Nucleotide Sequencing and Phylogenetic Analysis

**Figure 2:**
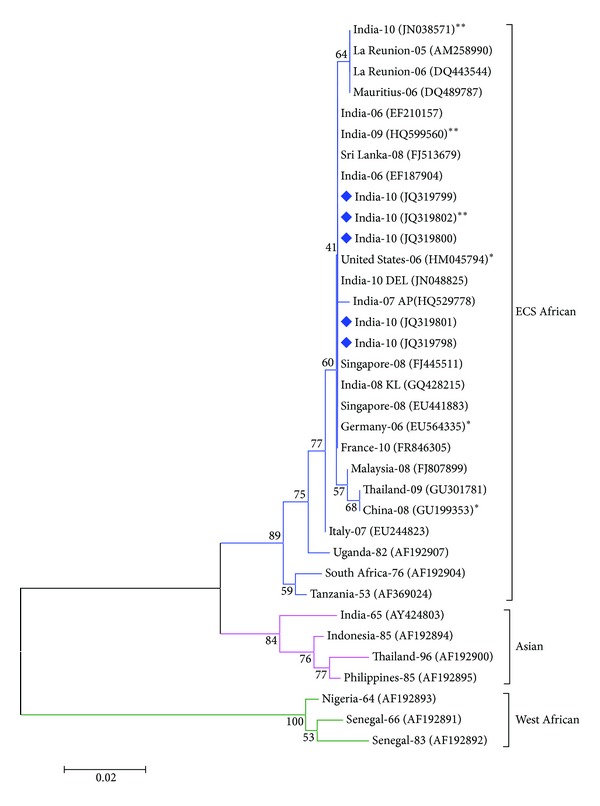
Phylogenetic tree among CHIK viruses generated by neighbor-joining method based on the partial nucleotide sequence of E1 gene. Each strain is abbreviated with the country of origin and last two digits of the year of isolation, followed by GenBank accession number in parenthesis (). The CHIKV sequenced in this study are marked with solid diamond (◆). Bootstrap values are indicated at the major branch points. * indicates the sequence of CHIKV from imported cases and ** indicates the sequences of CHIKV isolated from mosquito hosts.

The nucleotide sequencing of the partial E1 gene (457 nucleotides) of five CHIKV was determined in this study. These sequences were submitted to GenBank and Accession no. JQ319798–JQ319802 was assigned. These sequences were compared with twenty-nine other geographically diverse CHIKV isolates. The alignment did not reveal any nucleotide deletions or insertions. The phylogenetic analysis classified all the 35 CHIKV into three distinct genotypes. The dendogram revealed that all the five CHIKV strains from this outbreak belong to the ECS African genotype (Figure 2). This genotype is represented by large number of recent isolates from La Reunion (2005-06), Mauritius (2006), Sri Lanka (2008), Singapore (2008), Malaysia (2008), Thailand (2008), Italy (2007), and France (2010), in addition to number of imported cases in Germany (2006), United States (2006), and China (2008). The root of this genotype is represented by older African CHIKV isolates from Tanzania (1953), South Africa (1976), and Uganda (1982). However, the earlier Asian isolates from India, Indonesia, Philippines, and Thailand (1965–1996) were found to belong to the Asian genotype. The CHIKV isolates from Senegal and Nigeria (1964–1983) were grouped into the West African genotype.

## 4. Discussion

Chikungunya has emerged as a major public health problem in many tropical countries of Africa and Asia. Off late, it is considered as a potential threat to even temperate countries in Europe and the Americas [17]. The geographical expansion of *Aedes albopictus* to temperate zone makes these areas as higher risk. The recent local cases in Italy and France highlighted the epidemic potential of CHIKV in temperate regions [18, 19]. The explosive reemergence of Chikungunya with more than 4 million cases in India alone since 2006 is an international medical concern. Many Indian states continued to witness the infection and it is also reported from newer geographical area. In addition to the novel African genotype, the lack of herd immunity among population is often cited as the primary reason for its rapid spread across India [14].

The present focal outbreak was reported, during October-November 2010, in and around Gwalior in northern India. The occurrence of Chikungunya in postmonsoon season is also reported from other parts of India [13, 20]. The favorable mosquitogenic condition during postmonsoon period is primarily responsible for the rapid spread of Chikungunya. In spite of extensive attempt to control the mosquito population, it is far from realization.

Historically, the Chikungunya infection was reported to cause primarily mild infections in humans. However, the recent outbreaks witnessed wide array of symptoms among patients with unprecedented morbidity and mortality [1, 21]. A detailed molecular investigation was carried out on the clinical samples and the mosquito pools to ascertain the etiology of the outbreak. The RT-PCR technique has been successfully utilized for confirming Chikungunya in the acute phase of infection. The presence of CHIKV RNA in both clinical and mosquito samples by Chikungunya specific RT-PCR confirmed the etiology. The identification of CHIKV in *Aedes aegypti* pools confirms it as the primary vector of Chikungunya virus in northern India. Subsequently quantitative RT-PCR was carried out to determine the viral load in the samples. The CHIKV RNA load was found to range from 10^2.50^ to 10^6.76^ copies/mL. The one additional positive sample in qRT-PCR compared to end-point RT-PCR is due to lower RNA load (10^2.5^ copies/mL) in that sample. The higher sensitivity of qRT-PCR over end-point RT-PCR has already been widely reported [21, 22]. The higher viremia is also responsible for the rapid spread of infection in a community through mosquito vector [22, 23].

The phylogenetic analysis clearly classified the CHIKV from the present study to ECSA genotype. It formed a close branch along with large number of recent Indian CHIKV isolates, confirming the continued circulation of ECSA genotype viruses. The ECSA genotype is rapidly spreading in different parts of tropics and replacing the earlier circulating genotypes.

The detailed molecular investigation of both clinical and mosquito samples led to identification of ECSA genotype of CHIKV as the etiology of the present outbreak. This study also confirmed *Aedes aegypti* as the primary vector of Chikungunya virus in northern India. This ECSA genotype is attributed to prolonged chronic arthritis with fatal outcome on many instances. The recent evidence of rapid adaptation of this virus into mosquito population in temperate region points towards an alarming scenario in future. The persistence of Chikungunya virus and regular occurrence of outbreaks in south Asia is a major cause of concern, which requires continuous monitoring of the viral circulation in both endemic and non-endemic areas.

## Figures and Tables

**Table 1:** Signs and symptoms of Chikungunya infected patients.

Sign and symptoms	Number (percentage) of patients
Fever	52 (100)
Joint pain	42 (80)
Chill	32 (62)
Headache	16 (30)
Abdominal pain	4 (7.69)
Anorexia	9 (17.3)
Nausea	6 (11.53)

**Table 2:** Quantification of Chikungunya viral RNA in clinical samples and mosquito pools by qRT-PCR.

Sample ID*	Quantification of CHIKV RNA (copies/mL)	Ct value
DRDE10_GWL1	10^2.644^	28.12
DRDE10_GWL2	10^2.769^	27.72
DRDE10_GWL3	10^2.823^	27.55
DRDE10_GWL4	10^4.332^	22.73
DRDE10_GWL5	10^2.769 ^	27.72
DRDE10_GWL6	10^2.904^	27.29
DRDE10_GWL7	10^2.863 ^	27.42
DRDE10_GWL8	10^2.716^	27.89
DRDE10_GWL9	10^2.506 ^	28.56
DRDE10_GWL10	10^2.819^	27.56
DRDE10_GWL11	10^2.754 ^	27.77
DRDE10_GWL12	10^6.761 ^	14.97
DRDE10_GWL13	10^3.712 ^	24.71
DRDE10_GWL14	10^2.722^	27.87
DRDE10_GWL15	10^5.766^	18.15
DRDE10_GWL16	10^6.492 ^	15.83
DRDE10_GWL1M	10^3.270 ^	26.12
DRDE10_GWL2M	10^2.910^	27.27

*The sample ID with a suffix M refers to the mosquito samples.
